# The Third International Genomic Medicine Conference (3rd IGMC, 2015): overall activities and outcome highlights

**DOI:** 10.1186/s12864-016-3085-4

**Published:** 2016-10-17

**Authors:** Muhammad Abu-Elmagd, Mourad Assidi, Ashraf Dallol, Abdelbaset Buhmeida, Peter Natesan Pushparaj, Gauthaman Kalamegam, Emad Al-Hamzi, Jerry W. Shay, Stephen W. Scherer, Ashok Agarwal, Bruce Budowle, Mamdooh Gari, Adeel Chaudhary, Adel Abuzenadah, Mohammed Al-Qahtani

**Affiliations:** 1 Centre of Excellence in Genomic Medicine Research, King Abdulaziz University, Jeddah, Kingdom of Saudi Arabia; 2 Centre of Innovation in Personalized Medicine, King Abdulaziz University, Jeddah, Kingdom of Saudi Arabia; 3 Department of Cell Biology, University of Texas Southwestern Medical Center, Dallas, USA; 4 McLaughlin Centre and The Centre for Applied Genomics, Hospital for Sick Children and University of Toronto, Toronto, Canada; 5 Center for Reproductive Medicine, The Cleveland Clinic, Cleveland, USA; 6 Institute of Applied Genetics, Department of Molecular and Medical Genetics, University of North Texas Health Science Center, 3500 Camp Bowie Boulevard, Fort Worth, TX USA

## Abstract

The Third International Genomic Medicine Conference (3^rd^ IGMC) was organised by the Centre of Excellence in Genomic Medicine Research (CEGMR) at the King Abdulaziz University, Jeddah, Kingdom of Saudi Arabia (KSA). This conference is a continuation of a series of meetings, which began with the first International Genomic Medicine Conference (1^st^ IGMC, 2011) followed by the second International Genomic Medicine Conference (2^nd^ IGMC, 2013). The 3^rd^ IGMC meeting presented as a timely opportunity to bring scientists from across the world to gather, discuss, and exchange recent advances in the field of genomics and genetics in general as well as practical information on using these new technologies in different basic and clinical applications. The meeting undoubtedly inspired young male and female Saudi researchers, who attended the conference in large numbers, as evidenced by the oversubscribed oral and poster presentations. The conference also witnessed the launch of the first content for *npj Genomic Medicine*, a high quality new journal was established in partnership by CEGMR with Springer Nature and published as part of the Nature Partner Journal series. Here, we present a brief summary report of the 2-day meeting including highlights from the oral presentations, poster presentations, workshops, poster prize-winners and comments from the distinguished scientists.

## Introduction

Given the resounding success of the Second International Genomic Medicine Conference (2^nd^ IGMC) and the publication of its outcome [1], the Center of Excellence in Genomic Medicine Research (CEGMR) continued its trend of open science discussion and organized the Third International Genomic Medicine Conference (3^rd^ IGMC, 30^th^ November to 1^st^ December 2015) (http://igmc-cegmr.org/Default.aspx) at the King Faisal Convention Hall, King Abdulaziz University (KAU). The 3^rd^ IGMC provided a focused and in depth view of the latest research findings relating to cancer, genetics, genomic medicine, reproductive medicine, ageing, metagenomics and metabolomics, personalized genomics, biobanking, bioinformatics, along with many other interesting topics. At least 500 leading international researchers, renowned national scientists including those from KAU dedicated scientific faculty, participated proactively presenting their latest research findings. All the recognized universities and research organizations in Saudi Arabia including CEGMR, Center of Innovation in Personalized Medicine (CIPM), King Fahd Medical Research Center (KFMRC) and King Abdulaziz City for Science and Technology (KACST) participated. The following countries were represented: Canada, Egypt, Germany, Japan, Qatar, Spain, Switzerland, Tunisia, Turkey, United Kingdom (UK), and United States of America (USA).

One of the major events of the meeting was the announcement and launch of the first issue of the *npj Genomic Medicine*, a journal that was established by CEGMR in partnership with the Nature Publishing Group (NPG). In addition, a significant number of private companies attended the conference and took advantage of the meeting to present their state-of-the-art products, and services supporting genome research. Workshops (for all participants and others for local training endeavors) were organized over 3 days following the 2-day symposium. Here, we report the overall events and activities of the meeting including oral presentations, poster sessions, workshops, poster prize winners and comments from highly distinguished guest speakers about the meeting.

### 3^rd^ IGMC 1^st^ day, opening and welcoming session

On 30^th^ of November, the conference began symbolically with an opening ceremony and welcome reception proceeding with the recitation of Holy Quran. The session was chaired by Professor Adeel Chaudhary, Deputy Director at CEGMR and Vice Dean of the Faculty of Applied Medical Sciences, KAU. On the stage, the Chief Guest, His Highness Vice President Professor Adnan Zahid and the Director of CEGMR, Professor Mohammed Al-Qahtani were seated to accord the occasion. A large number of foreign delegates, guests of honor, scientists, faculty and participants from a variety of institutes were in attendance. Professor Mohammed Al-Qahtani, gave a welcome address (Table 1) and recalled the interdepartmental, regional and international collaborations that have been fostered since the inception of the center and how notable progress has been achieved and sustained as a result of these collaborations. As a mark of respect to the University, Professor Al-Qahtani presented a memento to Professor Adnan Zahid who obliged by accepting it. Professor Al-Qahtani presented Dr. Abdulaziz Al-Swailem, Vice President, King Abdulaziz City for Science and Technology (KACST), Riyadh, KSA who then delivered an important speech in which he briefed the attendees on the launch of the second phase of the next 5 year KACST research plan (Maarifah Two) (Table 1). In addition, further funding opportunities were presented by Dr. Omar El-Farouk, the Director of Qatar National Research Fund (QNRF), Qatar Foundation, Doha, Qatar. The attendees found this talk, in particular, a golden opportunity to apply for funding outside Saudi Arabia, which would be an excellent vehicle for networking amongst researchers working in Gulf countries.

**Table 1:** 3^rd^ IGMC 1^st^ Day oral talks, opening and welcoming session

Session I	
Opening and welcoming session	
Chairperson: Professor Adeel Chaudhary	
09:00–09:15	Professor Mohammed Al-Qahtani: Director of Centre of Excellence in Genomic Medicine Research, King Abdulaziz University, Jeddah, Kingdom of Saudi Arabia
	Title: Welcoming and Opening the 3rd IGMC
09:15–09:25	Professor Abdurrahman Alyoubi: President of King Abdulaziz University, Jeddah, KSA
	Title: KAU and its Role in Supporting the IGMC
09:25–09:35	Dr. Abdulaziz Al-Swailem: Vice President, King Abdulaziz City for Science and Technology, Riyadh, KSA
	Title: KACST and Prospects of Research in Saudi Arabia, Maarifah-2
09:35–09:50	Dr. Omar El Farouk Boukhris: Director, Qatar National Research Fund, Qatar Foundation, Doha, Qatar
	Title: Funding Opportunities at QNRF
09:50–10:05	Dr. Nazeeh Al-Othmany: Saudi FDA, KSA
	Title: Helping Research in Saudi Arabia
10:05–10:20	Dr. Magdalena Skipper: npj Executive Editor, Nature Publishing Group, United Kingdom
	Title: CEGMR & Nature Partnership

The 3^rd^ IGMC meeting, for the first time, invited a senior member (Dr. Nazeeh Al-Othmany) from the Saudi Food and Drug Administration (SFDA) who presented a detailed view of the workings of the organization, its aims and how all members of the SFDA organization are continuously trying to help and facilitate the mission of scientists at the research centers within Saudi Arabia. The welcoming session ended with a presentation by Dr. Magdalena Skipper, the npj Executive Editor, NPG, UK who presented the model of the partnership between the NPG and CEGMR, which has led to establishment of the first npj journal in the Middle East, npj *Genomic Medicine* (http://www.nature.com/npjgenmed/). This fruitful partnership between NPG and KAU has led to establishment of a second npj journal, npj *Climate and Atmospheric Science*, http://www.nature.com/npjclimatsci/. Later, the Editor-in-Chief Dr. Stephen Scherer of npj *Genomic Medicine* announced the launch of the first issue of the journal. After Dr. Skipper’s talk the proceedings of the conference were declared open and all attendees settled to scientific business.

### 3^rd^ IGMC 1^st^ day, session I: genomics of cancer, aging and complex diseases

The second session on Day 1 was dedicated mainly to “Genomics of Cancer, Ageing and Complex Diseases” (Table 2). This session was chaired by the distinguished Professor Angel Carracedo, a prominent clinician scientist affiliated with KAU, Professor Adnan Merdad (School of Medicine, KAU) and Professor Abdelbaset Buhmeida (CEGMR, KAU). The first talk was the keynote lecture given by Professor Jerry W. Shay from the Department of Cell Biology, University of Texas Southwestern Medical Center, Dallas, USA on “Ageing and Cancer: Are Telomeres and Telomerase the Connection?” Professor Shay discussed the regulation over long distances of genes by telomere length. He elucidated his group’s discovery using 3D Co-FISH and a modified method of chromosome capture followed by high-throughput sequencing (Hi-C) to identify a significant number of genes within the first 10 Mb distal to the telomeres on many chromosomes regulated by telomere length. Professor Shay showed that the human telomerase reverse transcriptase (TERT) locus was also linked with looped chromatin structures in cells with long telomeres but was reduced in cells with shorter telomeres. He termed telomere length dependent regulation of gene expression as telomere position effects over long distances (TPE-OLD). He also showed that gene expression was regulated by telomere length long before telomeres become short enough to initiate a DNA damage response. These findings demonstrated a novel function for telomeres and also suggested a new mechanism for how progressive telomere shortening might affect human age associated disease initiation and progression.

**Table 2:** 3^rd^ IGMC, 1^st^ Day, Session II

Session II	
Genomics of cancer, aging and complex diseases	
Chairpersons: Professor Adnan Merdad, Professor Angel Carracedo and Professor Abdelbaset Buhmeida	
10:40–11:20	Keynote Speaker, Professor Jerry W. Shay: Director, Cancer Biology, Department of Cell Biology, University of Texas Southwestern Medical Center, Dallas, USA
	Title: Aging and Cancer: Are Telomeres and Telomerase the Connection?
11:20–11:45	Professor Albert J. Fornace Jr.: Director, Waters Center of Innovation for Metabolomics; Molecular Cancer Research Chair, Lombardi Comprehensive Cancer Center; Georgetown University, USA
	Title: Integration of Metagenomics and Metabolomics in Gut Microbiome Research
11:45–12:15	Dr. Deema Hussein: King Fahd Medical Research Centre, King Abdulaziz University, KSA
	Title: A Unique Integrated System to Discern Pathogenesis of Central Nervous System Tumors
12:15–12:35	Professor Stephen Scherer: Director of the McLaughlin Centre and Centre for Applied Genomics, Hospital for Sick Children and University of Toronto, Toronto, Canada
	Title: A Genomic Basis for Autism Spectrum and Related Disorders
12:35–12:50	Professor Nobutaka Hirokawa: Departments of Cell Biology and Anatomy, Graduate School of Medicine, University of Tokyo, Japan
	Title: KIF2A Regulates Postnatal Axons and its Deficiency causes Malformation of Cortical Development and Epilepsy
12:50–13:05	Dr. Heba Alkhattabi: Centre of Excellence in Genomic Medicine Research, KAU, Jeddah, Saudi Arabia
	Title: RPL27A, a Novel Target for miR-595 and its Contribution to Myelopoiesis via Ribosomal Dysgenesis

The keynote lecture was followed by a presentation by Professor Stephen Scherer from the Centre for Applied Genomics, Hospital for Sick Children and University of Toronto, Canada on “Genomic basis for autism spectrum and related disorders”. He explained the substantial contribution of non-coding variants to the aetiology of autism spectrum disorders (ASD) and highlighted the significance of using whole genome sequencing (WGS) for comprehensive genetic analyses. A novel observation shared was an increased frequency of *de novo* mutations near to *de novo* copy number variations residing on the maternal chromosome. He also discussed how to integrate epigenomic data with whole genome sequence data and interpret complex data through clustering approaches. He further emphasized the importance of performing WGS of 10,000 individuals with ASD and their family members and applying this data to diagnostic and drug treatment design [2].

Professor Albert Fornace from the Lombardi Comprehensive Cancer Center, Georgetown University, USA then spoke on “Integration of metagenomics and metabolomics in gut microbiome research”. Professor Fornace presented his research group’s various discoveries using metagenomics and metabolomics strategies to understand the correlative nature of the microbe/host co-metabolism to distinguish the regulatory functions of microbiota in preserving the health of the host. He further explained the potential of host/microbial interaction studies to define causality, response to treatment, risk prediction, and keystone species to exploit as probiotics in diseases such as inflammatory bowel disease (IBD) or colonic inflammation (colitis) triggered by immune-radiotherapy.

Professor Nobutaka Hirokawa from the Departments of Cell Biology and Anatomy, and Molecular Structure and Dynamics, University of Tokyo, Japan presented interesting talk titled “KIF2A regulates postnatal axons and its deficiency causes malformation of cortical development and epilepsy”. He presented his findings on the importance of Kinesin-13 super family protein 2A (KIF2A), an ATP-dependent microtubule destabilizer, in the development of precise neuronal circuits, prenatal cell survival, migration and the elongation of axons. He further described various studies to decipher the role of KIF2A in neuronal development using tamoxifen-inducible conditional KIF2A knockout mice. He showed that the loss of KIF2A led to hippocampal sclerosis, dentate mossy fiber (MF) sprouting, and recurrent excitatory circuits resembling but different from temporal lobe (TLE) and these phenotypes developed without excitation. More importantly, he showed that the conditional KIF2A granule cells displayed failed axon/dendrite differentiation, and developed multiple short axons throughout the entire molecular layer.

Then Dr. Deema Hussain from KFMRC, KAU gave a presentation on “A unique integrated system to discern pathogenesis of central nervous system tumours”. Her presentation focused on the establishment of a unique integrated system to facilitate the analysis of multiple bio-parameters of distinct central nervous system tumours (CNST) such as meningiomas, astrocytomas, oligodendrogliomas, medulloblastomas and glioblastomas. She explained about generating cell lines from CNST for further genetic, biochemical, cytological and pharmacological analysis. She also elaborated further on the importance of gene expression profiling of uncultured tumour samples to enable identification of several novel prognostic markers for meningiomas, including the recently associated cancer stem cell marker called anterior gradient protein 2 homolog (AGR-2). Dr. Deema concluded that the integrated analysis approach was essential for the development of safe, efficacious and potent targeted therapeutic modalities for CNST.

The final presentation of the second session on Day 1 was delivered by Dr. Heba Alkhattabi from CEGMR on “RPL27A, a novel target for miR-595 and its contribution to myelopoiesis via ribosomal dysgenesis”. She discussed the identification of several targets for miR-595 including a large ribosomal subunit RPL27A using a novel method. Dr. Alkhattabi explained that the down regulation of RPL27A triggered p53 activation, apoptosis and inhibited proliferation. More significantly, she showed that p53-independent effects were observed secondarily to a reduction in the ribosome subunit 60s with associated ribosome dysgenesis. In addition, RPL27A overexpression did not influence the p53 mRNA levels but enhanced proliferation. RPL27A knockdown in normal CD34+ cells specially inhibited erythroid proliferation and differentiation. Dr. Alkhattabi concluded that haploinsufficiency of miR-595 in Myelodysplastic Syndromes (MDS) patients with - 7/del 7q may contribute to disease pathogenesis via RPL27A modulation since miR-595 was significantly down regulated in these patients with −7/-7q anomalies.

The final scientific session of the first day (Session III) was on "Genomics applications towards personalized medicine" (Table 3) with all presentations focused on the theme of how the latest advancements in genomics could translate to personalization, also referred to as the precision or personalized medicine. Professor Emmanouil Dermitzakis from the Department of Genetic Medicine and Development, University of Geneva Medical School, Switzerland highlighted the genotype-tissue expression (GTEx) project, where multiple 'non-diseased' tissue samples collected from recently deceased human donors could be used for analysis across tissues and individuals. The patterns of transcriptome variations across individuals and tissues and their association with human phenotypes can be analyzed using GTEx data. Furthermore, quantification of gene expression by RNA-sequencing on GTEx samples had high concordance with microarray based quantification, thus indicating the value of the samples. Recent advances in tumor biology will enable personalization of cancer therapeutics with reliance on information from the International Cancer Genome Consortium (ICGC), The Cancer Genome Atlas (TCGA) and the Cancer Genome Project (CGP) as well as the understanding of the oncogenic drivers, the genes involved in disease progression, the identification of biomarkers and the development of drugs for specific targets.

**Table 3:** 3^rd^ IGMC, 1^st^ Day, Session III: Genomics applications towards personalized medicine

Session III	
Genomics applications towards personalized medicine	
Chairpersons: Professor Jerry Shay, Professor Jaudah Al-Maghrabi and Dr. Muhammad Abu-Elmagd	
14:35–14:55	Professor Emmanouil Dermitzakis: Department of Genetic Medicine and Development, University of Geneva Medical School, Switzerland
	Title: Population and Personalized Genomics to Understand Disease and Personal Biology
14:55–15:15	Professor Anne Goodeve: Department of Cardiovascular Science, University of Sheffield, Medical School, UK
	Title: Experience of Next-Generation DNA Sequence Analysis in a UK Genetics Laboratory
15:15–15:45	Dr. Ashraf Dallol: Centre of Excellence in Genomic Medicine Research and CIPM, KAU, Jeddah, KSA
	Title: Genomics of Colon Cancer From Saudi Arabia

Along similar lines, Professor Anna Goodeve from Sheffield Diagnostic Genetic Service, Sheffield Children's NHS Foundation Trust, UK, shared her experience on how next generation sequencing (NGS) and gene panels have extended diagnostic capability in analyzing blood related disorders. The haemostasis and platelet gene panel which included 13 genes and another panel consisting of 16 genes that are currently known to be associated with Fanconi's anaemia (FA) were sequenced using the Illumina MiSeq or HiSeq. Dr. Goodeve’s results revealed that the haemostasis and platelet panel enabled identification of pathogenic candidate mutations in 62 % of the patients. The FA panel led to the identification of biallelic mutations (33 %), heterozygous mutations (13 %), homozygous mutations (13 %), other gene mutations (7 %) and no mutations (33 %). She concluded that the use of NGS provides a single laboratory workflow for analysis of gene panels for different disorders and considerably reduces time to achieve a definitive diagnosis.

In the final presentation of this session, Dr. Ashraf Dallol, from the CIPM/CEGMR at KAU, explained the usefulness of targeted sequencing of cancer mutational hotspots in combination with the Ion Torrent Personal Genome Platform in a cohort of colorectal cancer (CRC) cases from Saudi Arabia. CRC is a heterogeneous disease influenced by genetic and epigenetic variations acting as passengers or drivers of the tumor. Dr. Dallol showed that analysis of the mutational status of 2,800 catalogued somatic mutations in cancer (COSMIC) mutations of 50 oncogenes and tumor suppressor genes in CRC would enable understanding the genetic background for this cohort and thus pave the way to precision medicine (PM).

### 3^rd^ IGMC 2nd day, session I, biobanking in the post-genomic era

Since the completion of the Human Genome Project in 2003, a significant increase in the number of Biobanks (also called Biorepositories including their improved design, scale and governance) has been observed worldwide. The main aim of establishing these banks was to support effective genomic research and personalized healthcare. Nowadays, Biobanks are considered as the main core around which OMICs research and advanced clinical applications are carried out to drive PM [3]. However, a major PM challenge research community currently facing is the need for a large scale of high-quality and fully annotated bio- specimens. To address such challenge, thousands of biobanks in developed countries have been established and are currently working towards harmonizing their practices internationally [4]. However, this big number of biobanks established in the developing countries needs to establish crucial resources to overcome lack of knowledge, awareness, and practice (KAP) in addition to other technical, operational, ethical and regulatory challenges.

Therefore, the 3^rd^ IGMC “Biobanking in the Post-Genomic Era” session (Table 4) was aimed at raising the level of knowledge and awareness of the research community and public within Saudi Arabia, the Gulf region and at the international level in order to draw attention to the importance of this critical resource. During the session, the quantity and quality of biospecimens were highlighted by both international (Professor Jim Vaught) and national (Professor Abdelbaset Buhmeida) speakers. They demonstrated that this issue is fundamental in order to produce effective diagnostic tools and tailor customized drugs to enhance the clinical service offered to patients. The need for additional high quality and fully annotated biospecimens processed using Standard Operating Procedures (SOPs) was also highlighted as one of the top priorities of biomedical research in order to deliver more accurate downstream diagnostic test results. Dr. Ibrahim Alabdulkareem from the National Guard Health Affairs, Riyadh, KSA and Professor Jaudah Al-Maghrabi from Pathology Department, College of Medicine, KAU, Jeddah, KSA strongly emphasized this issue. The Biobank session witnessed extensive discussion from the audience and this was marked by motivation from both patients who attended this session to donate their biospecimens and scientists who requested support and guidelines from CEGMR biobanks.

**Table 4:** 3^rd^ IGMC, 2^nd^ day, Session I: Biobanking in the post-genomic era

2^nd^ day, Session I	
Biobanking in the post-genomic era	
Chairpersons: Professor Stephen Scherer, Dr. Mourad Assidi and Dr. Farid Ahmed	
09:00–09:30	Professor Jim Vaught: President, ISBER, Editor-in-Chief Biopreservation and Biobanking, International Prevention Research Institute, Maryland, USA
	Title: Current Perspectives in International Biobanking
09:30–09:45	Dr. Ibrahim Alabdulkareem: Chairman of Medical Genomic Research Department, King Abdullah International Medical Research Center, King Abdulaziz Medical City, National Guard Health Affairs, Riyadh, KSA
	Title: Saudi Biobanks: Importance, Implications and Opportunities
09:45–10:00	Professor Abdelbaset Buhmeida: Centre of Excellence in Genomic Medicine Research, KAU, Jeddah, KSA
	Title: The CEGMR Biobanking Unit: Achievements, Challenges and Future Plans
10:00–10:15	Professor Jaudah Al-Maghrabi: Pathology Department, College of Medicine, KAU, Jeddah, KSA
	Title: Tissue Microarray Technique: A powerful Adjunct Tool for Molecular Profiling of Solid tumors

### IGMC 2^nd^ day, session II: bioinformatics applications in genomic medicine

The continuous development of new OMICs technologies has led to generation of unprecedented large data sets. Handling and analysis of these OMICs data to extract meaningful and clinically actionable targets are serious challenges [5]. Additional approaches are required to achieve integrative analysis of heterogeneous data. That is to tailor meaningful predictions and associations of the data in order to better understand the molecular pathology of diseases to improve targeted treatment [6, 7]. With the integration of OMICs in modern healthcare, the 3^rd^ IGMC dedicated a session to “Bioinformatics applications in genomic medicine” (Table 5). Given the complexity of cancer, the session started with a presentation by Professor Sudhir Kumar, Department of Biology, Temple University, Philadelphia, USA, who showed an analysis of cancer heterogeneity assessed from an evolutionary prospect and demonstrated its use in tracing the origins, patterns, and evolutionary relationships of homologous sequences of cancer clones (clonotypes) to overcome the hurdle of cancer heterogeneity for diagnosis, prognosis and therapeutics [8].

**Table 5:** 2^nd^ day of the 3^rd^ IGMC, Bioinformatics applications in genomic medicine

Session II	
Bioinformatics applications in genomic medicine	
Chairpersons: Professor Jim Vaught and Dr. Ashraf Dallol	
11:35–11:55	Professor Sudhir Kumar: Director, Institute for Genomics and Evolutionary Medicine, Carnell Professor, Department of Biology, Temple University, Philadelphia, USA
	Title: Phylomedicine of Tumors
11:55–12:15	Professor Angel Carracedo: Director of the Galician Foundation of Genomic Medicine, Professor of Legal Medicine Santiago de Compostela University, Spain
	Title: Cancer Genetics: From GWAS to the Discovery of Rare Variation
12:15–12:35	Professor Hossam Faheem: Computer Systems International Consultant; HPC Project Manager, Fujitsu Technology Solutions International
	Title: The Supercomputer Facility “AZIZ” at KAU: Utility and Future Prospects
12:35–12:50	Dr. Sajjad Karim: Centre of Excellence in Genomic Medicine Research, KAU, Jeddah, KSA
	Title: E-GRASP: A Resource for Exploring Outcomes of the Genome-Wide Association Studies through the Lens of Interspecific Evolutionary History

Moving from the genome-wide association study (GWAS) scale to target specific rare and disease-specific variations was another major focus of the conference delivered by Professor Angel Carracedo from Legal Medicine Santiago de Compostela University, Spain. He showed that moving from GWAS-driven associations to causality is a key turning point towards personalized medicine. However, a further filtering step of the large number of associations revealed by GWAS with reduced heritability is necessary to identify causality or susceptibility targets. Professor Carracedo suggested new targeted re-sequencing of GWAS association targets in order to refine both the scale and mapping of the genomic hits. These identified hits would provide clues to decrypting the molecular pathways.

Professor Carracedo’s talk was followed by that of Professor Hossam Faheem from Fujitsu Technology Solutions International, Japan who showed that several factors including computer science, medical informatics, Biobanking LIMS (Laboratory Information Management System), bioinformatics, biostatistics and OMICs datasets should be joined to tailor a clinical integrative analysis workflow of heterogeneous datasets in order to drive the current transition from OMICs-based science to OMICs-based healthcare. He showed that advanced computational tools such as the supercomputing facilities with superior memory to accommodate parallel and computationally intensive applications’ needs offer excellent solution which are now implemented at KAU (named as AZIZ supercomputer facility).

The bioinformatics session ended with a presentation by Dr Sajjad Kareem from CEGMR who highlighted developing a new web application called E-GRASP which allows: (i) SNPs view including information about SNPid, PMID, P-value, chromosome, position, number of studies and phenotypes for each SNPs, (ii) studying view; which explains unique SNPs replication in each studies and phenotypes and (iii) evolutionary view which describes evolutionary information including E-value for SNP phenotype association, E-rank of the evolutionary rate and time span of the position retrieved from the “E-rank Web Server” for each SNP. Dr. Karim concluded that E-GRASP is a more representative database with additional information of SNPs replication and evolutionary scores, facilitating the comprehensive qualitative and quantitative analysis.

### IGMC 2nd day, session III: reproductive medicine in the 21^st^ century

The investigation of disease aetiologies requires a complex and multidimensional approach. Sufficient understanding of these diseases origins and susceptibility is an important prerequisite. Thus, investigation of germline genomic instability (mutations and epi-mutations) and its role in disease heritability and/or susceptibility during reproductive and embryonic phase will provide clues about diseases’ origin and relevant causative genomic variations. The Reproductive Medicine in the 21^st^ Century Session at the 3^rd^ IGMC was dedicated to highlight the revolution in reproductive and developmental medicine due to the OMICs-based application that can offer successful and cost-effective solutions.

Dr. Aisha Elaimi from the CEGMR/KAU (Table 6) highlighted how an experimental animal model system, such as the Embryoscope™, can be used to assess the developmental stages of an embryo directly from two cell to blastocyst stages in the presence or absence of pharmacological agents. Such an experimental system can be utilized to have a better understanding of the impact of blastulation rate and aneuploidy or DNA fragmentation, which in turn can lead to personalized therapeutic applications.

**Table 6:** 2^nd^ day of the 3^rd^ IGMC, Reproductive medicine in the 21^st^ century

Session III	
Reproductive medicine in the 21^st^ century	
Chairpersons: Professor Taha Abo Almagd and Dr. Gauthaman Kalamegam	
14:20–14:40	Dr. Aisha Elaimi: Center of Innovation in Personalized Medicine, King Abdulaziz University, Jeddah, KSA
	Title: The effect of GM-CSF on murine embryo development, aneuploidy, DNA fragmentation and morphokinetic parameters analyzed using Embryo Scope
14:40–15:00	Dr. Serdar Coskun: King Faisal Specialist Hospital & Research Center, Riyadh, KSA
	Title: A New Look to Reproductive Medicine in The Era of Genomics
15:00–15:20	Professor Ashok Agarwal: Director, Center for Reproductive Medicine, The Cleveland Clinic, Cleveland, USA
	Title: New Research into the Causes of Male Infertility
15:20–15:40	Professor Eberhard Nieschlag: Emeritus Professor, Center for Reproductive Medicine and Andrology, University Hospital of Muenster, Germany
	Title: The Klinefelter Syndrome: Recent Progress

Dr. Serdar Coskun from King Faisal Specialist Hospital and Research Center, Riyadh, KSA addressed the issue of the OMICs scale applications and their impact in preimplantation genetic testing (PGT), where only one or a few cells are available for genomic analysis. He showed that the development of single cell whole genome amplification (scWGA) technologies enabled obtaining sufficient high-quality DNA from the initial minute starting material [9]. Dr. Coskun showed that PGT allowed the birth of a healthy baby who can potentially donate either the cord blood or haematopoietic stem cells to treat another ill patient.

Professor Ashok Agarwal, Center for Reproductive Medicine, The Cleveland Clinic, Cleveland, USA, focused his presentation on addressing new research into the causes of male infertility including proteomics which is the new frontier of research in male infertility as it allows the identification of numerous sperm-specific proteins. Prof. Agarwal concluded that proteomics provides a greater understanding of protein functions involved in sperm processes such as motility, capacitation, acrosome reaction and fertilization.

The reproductive medicine session wrapped up with a presentation by Professor Eberhard Nieschlag, Center for Reproductive Medicine and Andrology, University Hospital of Muenster, Germany, who focused on the Klinefelter Syndrome (KS) and its relationship with infertility. He showed that the incidence of KS in the general male population is 1:500 and among 23,000 patients of the Center for Reproductive Medicine and Andrology at the University of Muenster, Germany is 1:52, whereas in Saudi Arabia the incidence is 1:11 in selected patients with azoospermia or sperm counts <5 mill/ml. Professor Nieschlag’s presentation drew the attention to the serious impacts of the KS on Saudi infertility patients.

### Workshops

The scientific sessions of the 3rd IGMC were accompanied by eight workshops. These workshops offered the opportunity for local researchers to enhance their practical knowledge as well as to introduce the novice to the wide array of applications and cutting edge technologies available to researchers. The practical IGMC workshops covered a range of areas such as molecular cytogenetics in medicine, molecular genetics, flow cytometry in oncology, bioinformatics, high throughput DNA sequencing and microarrays. One additional workshop was introduced for the first time in the IGMC, the publication workshop, which was very well-received by the participants. Overall, the high demand for such wide range of training was demonstrated by the large number of applicants (*n* = 48) enrolling in the workshops. The venue for training was provided by CEGMR at KAU and many of the workshops were overseen and delivered by highly distinguished, international faculty who participated in the IGMC conference in a number of capacities. The success of the workshops program demonstrated the desire and enthusiasm of many local researchers to extend their knowledge in different fields and further develop their professional capabilities.

### 3^rd^ IGMC young scientists’ poster winners

The conference organizers recognized the importance of motivating young scientists and hence offered prizes for the best poster presentations. Nearly two hundred posters were displayed covering a wide range of research topics. A selected committee critically assessed the displays; posed questions to the participants; evaluate their explanations on the subject; and on the basis of a set of criteria, selected for the three top poster presentations. Each winner received a certificate and a monetary award. The first prize (5000 Saudi Riyals, SR) was awarded to Olfat Al-Harazi from the Department of Biostatistics, Epidemiology and Scientific Computing, King Faisal Specialist Hospital and Research Centre, Riyadh, KSA (Poster number 30) titled ‘’Breast Cancer Incidence and Genomic Signature in Young and Old Women in Saudi Arabia and Western Countries‘’. The second prize (4000 SR) went to Nezar Al-hebshi, Department of Preventive Dentistry, College of Dentistry, Jazan University, Jazan, KSA (Poster number 23) titled ‘’Mutational landscape of Arabian snuff-associated oral squamous cell carcinoma‘’. The third prize (SR 3000) was earned by Nada Salem from CEGMR/KAU, KSA (Poster number 105) titled: ‘’Integrated use of evolutionary information in GWAS reveals important SNPs in Asthma‘’.

### 3^rd^ IGMC scientific committee members

This committee consisted of nine members whose role was to carefully review the abstracts, make final decisions on the acceptance or rejection of submitted abstracts, and to decide which abstracts would be presented either as a poster or as an oral presentation. The members of the scientific committee are as follows:

1. Dr. Muhammad Abu-Elmagd
2. Dr. Ashraf Dallol
3. Professor Jerry W. Shay
4. Dr. Gauthaman Kalamegam
5. Dr. Mourad Assidi
6. Professor Abdelbaset Buhmeida
7. Dr. Farid Ahmed
8. Professor Stephen W. Scherer
9. Professor Bruce Budowle
10. Dr. Hans H. Schulten

Editing the 3^rd^ IGMC abstracts’ and articles’ supplements was led by Dr. Muhammad Abu-Elmagd who worked as the editor of both supplements. The 3^rd^ IGMC abstracts’ supplement was published in BMC Genomics (https://bmcgenomics.biomedcentral.com/articles/supplements/volume-17-supplement-6). The articles' supplement containing 16 peer-reviewed articles was published in a number of BMC journals including BMC Genomics, BMC Medical Genetics, BMC Cancer and BMC Structural Biology. The final decision on accepting and rejecting manuscripts was made by the Executive Editor of BMC Genomics.

### Thoughts about the 3^rd^ IGMC by eminent scientists

1. Guest keynote speaker Professor Jerry W. Shay: ‘’This was by far the most successful International Genomic Medicine Conference that was held in Jeddah, November 2015. There was a very large number of attendees that led to many discussions about science enabling many new collaborations. The invited speakers were excellent, covering diverse areas of genomics and human disease. The organization of the meeting was simply outstanding. Everything worked from registration, the overall program and most importantly the poster sessions. As one of the judges of the poster sessions, I had many conversations with young scientists. Their excitement about their work was contagious and will certainly have a future positive impact for science in the Kingdom of Saudi Arabia‘’.
2. The Director of the Centre for Applied Genomics, The Hospital for Sick Children and The Director of McLaughlin Centre for Molecular Medicine, University of Toronto, Canada, Professor Stephen Scherer: ‘’The meeting was memorable because all of the discussions were grounded in science and in data quality including interpretation of results. The session on Biobanking was very important since this can be done 'right' or it can be done 'wrong' and there needs to be scholarly discussion early on in decision-making. Perhaps, most importantly, I was energized by the excitement of the younger people participating at the meeting including many who traveled from remote regions of the Kingdom. There were many good lessons to be learned from the locals, national, and international participants‘’.
3. The Director of the American Center for Reproductive Medicine, Cleveland Clinic, USA, Professor Ashok Agarwal: ‘’I am pleased to share my impressions of the Third International Genomics Medicine Conference organized by the Center for Excellence in Genomic Medicine Research at King Abdulaziz University, Jeddah, Saudi Arabia in November 2015. The meeting was nicely organized in a pleasant venue, it had a large number of attendees and a galaxy of highly recognized speakers from many parts of the world. The keynote lectures went really well; there was lively discussion after these talks. The presentation by local team of researchers was of high quality and a good indicator of future developments in the field of genomic medicine. I came back with good memories of the excellent scientific discourse, opportunity to meet and listen to both local and international scientists and most importantly the warm hospitality of the local organizing committee‘’.

## Conclusions

The 3rd International Genomic Medicine Centre set a stage for the next round of planning and scientific investment in the area of individualized (precision) medicine in Saudi Arabia [10]. The active participation of female Saudi scientists was unprecedented. The conference witnessed the launch of the first issue of npj *Genomic Medicine*, a journal CEGMR/KAU has established in partnership with the Nature Publishing Group (NPG) that promises to expand publishing options in the Middle East and around the world. The conference workshops offered extensive training to the participants in a wide range of genomics tools and approaches. The take away message from most talks was that studying the population structure and health challenges confronting Saudi Arabia and by openly publishing the results would not only help people in this region, but also provide lessons for other countries around the world.

## Abbreviations

3rd IGMC: The Third International Genomic Medicine Conference
ART: Assisted reproductive technologies
ASD: Autism spectrum disorders
CEGMR: Centre of excellence in genomic medicine research
cfDNA: Cell-free DNA
CGP: Cancer genome project
CIPM: Center of innovation in personalized medicine
CNST: Central nervous system tumours
CRC: Colorectal cancer
FA: Fanconi's anaemia
FDA: Saudi Food and Drug Administration
GTEx: Genotype-tissue expression
GWAS: Genome-wide association study
Hi-C: High-throughput sequencing
HLA: Human leukocyte antigen
IBD: Inflammatory bowel disease
ICGC: International cancer genome consortium
KACST: King Abdulaziz City for science and technology
KAP: Knowledge, awareness, and practice
KAU: King Abdulaziz University
KFMRC: King Fahd Medical Research Center
KIF2A: Kinesin-13 super family protein 2A
KS: Klinefelter syndrome
KSA: Kingdom of Saudi Arabia
MDS: Myelodysplastic syndromes
NGS: Next generation sequencing
NPG: Nature Publishing Group
PGT: Preimplantation genetic testing
PM: Precision medicine
QNRF: Qatar National Research Fund
SCWGA: Single cell whole genome amplification
SOPs: Standard operating procedures
TCGA: The Cancer Genome Atlas
TERT: Telomerase reverse transcriptase
TLE: Temporal lobe
TPE: Telomere position effects
TPE-OLD: Telomere position effects over long distances
WGS: Whole genome sequencing
